# 芦可替尼治疗不同年龄分层骨髓纤维化患者的疗效及预后因素分析

**DOI:** 10.3760/cma.j.cn121090-20250113-00025

**Published:** 2025-08

**Authors:** 筱涵 刘, 媛 于, 拂蒙 阎, 晴 孟, 新文 姜, 庆莉 纪, 振一 刘, 月月 郑, 敏然 周, 赛 马, 春燕 陈

**Affiliations:** 1 山东大学齐鲁医院血液内科，济南 250012 Department of Hematology, Qilu Hospital of Shandong University, Jinan 250012, China; 2 威海市立医院血液内科，威海 264200 Department of Hematology, Weihai Municipal Hospital, Weihai 264200, China

**Keywords:** 骨髓纤维化, 芦可替尼, 年龄, 疗效, 基因突变, 预后, Myelofibrosis, Ruxolitinib, Age, Efficacy, Gene mutation, Prognosis

## Abstract

**目的:**

分析不同年龄分组的骨髓纤维化（myelofibrosis, MF）患者的临床特征、染色体核型及基因突变特点，比较芦可替尼治疗不同年龄分层MF患者的疗效、安全性及预后影响因素。

**方法:**

纳入2017年1月1日至2024年7月1日期间在山东大学齐鲁医院血液科接受芦可替尼治疗的188例MF患者，根据患者的诊断年龄分为≤55、56～65、>65岁三组，比较不同年龄组患者的临床特征、染色体及基因突变特点，评价疗效及安全性，利用Cox风险回归模型进行单因素和多因素分析明确影响总生存期的相关因素。

**结果:**

>65岁组基础合并症较多、治疗前症状负荷较重、白细胞计数较高、JAK2基因突变检出率及突变频率较高、CALR基因突变检出率较低，56～65岁组非驱动基因突变检出率较高。芦可替尼治疗后，≤55岁、56～65岁和>65岁组分别有50.9％（27/53）、43.5％（27/62）、45.5％（20/44）的患者获得左肋缘下可触及脾脏长度较基线减少≥50％（*P*＝0.720），54.0％（27/50）、60.3％（41/68）、66.7％（34/51）的患者获得MPN-10症状评分总分降低≥50％（*P*＝0.429）。最常见的血液学不良反应为贫血和血小板减少，非血液学不良反应为电解质紊乱、转氨酶升高和肺部感染。多因素分析结果显示，年龄增加、血红蛋白值降低、骨髓原始细胞比例≥1％、无JAK2突变、存在染色体核型异常、≥2个高分子风险突变、TP53突变阳性是接受芦可替尼治疗MF患者总生存期的独立危险因素。

**结论:**

不同年龄组MF患者的临床特征和基因突变存在异质性，但具有相似的芦可替尼疗效及不良反应发生率。

## Abstract (en)

**Objective:**

To explore differences in the efficacy and safety of ruxolitinib in patients with myelofibrosis by age and to identify prognostic factors by analyzing clinical features and characteristics of chromosomes and gene mutations.

**Methods:**

This study retrospectively analyzed 188 patients with myelofibrosis who received ruxolitinib in the Department of Hematology, Qilu Hospital, Shandong University from January 1, 2017, to July 1, 2024. According to age at diagnosis, the patients were divided into the middle-aged group（≤55 years）, young elderly group（56–65 years）, and elderly group（>65 years）. Clinical features, the characteristics of chromosomes and gene mutations, and the efficacy and safety of ruxolitinib treatment were compared across the three age groups. Independent factors influencing overall survival were identified through Cox proportional risk regression analysis.

**Results:**

Before treatment, the elderly group had more underlying comorbidities, a heavier symptom burden, higher leukocyte count, higher proportion and frequency of JAK2 mutations, and lower proportion of CALR mutations. The incidence of nondriver gene mutations was significantly higher in the young elderly group. After ruxolitinib treatment, the degree of reduction in spleen size did not differ significantly among the three groups. The length of the palpable spleen below the left costal margin reduced by more than 50％ from baseline in 50.9％（27/53）of the patients in the middle-aged group, 43.5％（27/62）in the young elderly group, and 45.5％（20/44）in the elderly group（*P*＝0.720）. No significant difference was observed among the three groups in the degree of reduction in Myeloproliferative Neoplasm Symptom Assessment Form（10-item version）score（*P*＝0.153）, with a reduction in total symptom score by more than 50％ achieved by 54.0％（27/50）, 60.3％（41/68）, and 66.7％（34/51）of the patients from the three groups, respectively（*P*＝0.429）. The most common hematological adverse events were anemia and thrombocytopenia, while the most common nonhematological adverse events were electrolyte disturbance, elevated transaminase activity, and pulmonary infection. Multivariate analysis indicated that in ruxolitinib-treated patients with myelofibrosis, poor overall survival was independently predicted by increased age, reduced hemoglobin, percentage of bone marrow blasts ≥ 1％, absence of JAK2 mutations, chromosomal abnormalities, ≥2 high-molecular-risk mutations, and TP53 mutations.

**Conclusion:**

Patients with myelofibrosis stratified by age exhibited heterogeneous clinical features and gene mutation profiles but similar efficacy of ruxolitinib treatment and occurrence of adverse events.

骨髓纤维化（myelofibrosis, MF）是骨髓增殖性肿瘤（myeloproliferative neoplasm, MPN）中预后最差的一种，临床特征包括脾肿大、全身症状和血细胞减少，估计中位总生存（OS）期为3.6～6.5年[1]–[2]。JAK2、CALR或MPL是MPN的已知驱动突变，>80％的原发性骨髓纤维化（PMF）患者携带ASXL1、TET2、EZH2、SRSF2、DNMT3A、U2AF1和IDH1/IDH2等其他髓系基因突变[3]。以往研究发现，年龄与MF患者等位基因负荷、非驱动突变及高分子风险（high molecular risk, HMR）突变的发生率和突变负荷相关[4]。芦可替尼（ruxolitinib）是首个靶向JAK1和JAK2突变的口服抑制剂，COMFORT-Ⅰ、COMFORT-Ⅱ及后续临床试验证实其对于MF患者具有缩脾、改善症状和延长OS期的作用[5]–[7]。随着人口老龄化加剧和人均寿命的延长，芦可替尼越来越多地应用于65岁以上老年患者。不同年龄段MF患者的临床特征及基因突变是否存在异质性、疗效及安全性是否存在差异，需要进一步探究。本研究对我院188例MF患者的临床数据进行了回顾性分析，旨在评估芦可替尼在不同年龄段MF患者中的疗效和安全性，探讨基因突变的特征及其对临床预后的影响。

## 病例与方法

一、病例

本项回顾性研究纳入2017年1月1日至2024年7月1日期间在山东大学齐鲁医院血液科接受芦可替尼单药或联合治疗的188例MF患者。所有患者均符合骨髓增殖性肿瘤WHO指南（2022版）和NCCN指南（2022版）的PMF、真性红细胞增多症后骨髓纤维化（PPV-MF）、原发性血小板增多症后骨髓纤维化（PET-MF）诊断标准。收集性别、年龄、体质性症状（体重下降、不能解释的发热、盗汗）、基础合并症、有无肝大、左肋缘下可触及脾脏长度、MPN-10评估表总分（MPN-SAF TSS）、血常规、骨髓原始细胞比例、外周血原始细胞比例、骨髓纤维化分级、染色体核型、驱动基因突变类型、非驱动基因突变（其中HMR突变包括ASXL1、IDH1/2、SRSF2、EZH2、U2AF1）、输血依赖性、骨髓纤维化亚型等基线特征，采用国际预后积分系统（IPSS）、动态国际预后积分系统（DIPSS）、动态国际预后积分系统加强版（DIPSS-Plus）、基于突变增强的国际预后积分系统（MIPSS-70）及MIPSS-70+2.0对患者进行预后评估和分组。本研究获得山东大学齐鲁医院医学伦理委员会审查通过（KYLL-202406-033-1）。

二、治疗方案

所有患者均接受芦可替尼单药或联合治疗，起始剂量根据血小板计数调整。

三、疗效评估

疗效评估指标包括左肋缘下可触及脾脏长度较基线减少≥35％和≥50％、MPN-10症状评分总分降低≥50％（TSS50）。

四、安全性评估

记录芦可替尼治疗期间出现的不良反应，根据美国国家癌症研究所常见不良反应术语评定标准（CTCAE）5.0版评估并分级。

五、随访

通过查阅门诊、住院资料以及电话、微信联系的方式进行随访，截止时间为2024年10月1日。OS期定义为自疾病明确诊断至因任何原因死亡或末次随访时间。中位随访时间27.8（15.1～42.8）个月。

六、统计学处理

数据分析和绘图采用SPSS 26.0、Graphpad prism 9.0、R 4.2.1软件。计量资料以中位数（四分位数间距）描述，分类资料以“频数（构成比）”描述。计量资料的组间比较采用单因素方差分析（ANOVA）和Bonferroni校正，分类资料组间比较采用卡方检验或Fisher确切概率法。生存分析采用Kaplan-Meier法，比较生存曲线的差异用Log-rank检验。采用Cox回归分析对疾病特征与OS的关系进行单因素及多因素分析。均以*P*<0.05为差异有统计学意义。

## 结果

一、临床特征

共纳入188例接受芦可替尼治疗的MF患者，中位年龄61（29～84）岁，以55、65岁为界限，将患者分为三组：≤55岁组57例（30.3％），中位年龄50（29～55）岁；56～65岁组76例（40.4％），中位年龄61（56～65）岁；>65岁组55例（29.3％），中位年龄69（66～84）岁。三组患者基础合并症、MPN-10症状评分总分、白细胞计数、骨髓纤维化分级≥2和驱动基因类型比较，差异均有统计学意义（*P*<0.05）。组间比较结果显示，>65岁组存在基础合并症的患者占比较高（与≤55岁组比较，*P*<0.001；与56～65岁组比较，*P*＝0.028）；>65岁组白细胞计数高于≤55岁组（*P*＝0.034）；>65岁组MPN-10症状评分总分较高（与≤55岁组比较，*P*＝0.003；与56～65岁组比较，*P*＝0.018）；56～65岁组骨髓纤维化分级≥2患者占比高于≤55岁组（*P*＝0.011）；>65岁组JAK2突变检出率较高（与≤55岁组比较，*P*＝0.029；与56～65岁组比较，*P*＝0.030），CALR突变检出率较低（与≤55岁组比较，*P*＝0.003；与56～65岁组比较，*P*＝0.011）。芦可替尼治疗前患者的临床特征见表1。

**表1:** 188例骨髓纤维化患者芦可替尼治疗前临床特征

临床特征	≤55岁组 （57例）	56～65岁组 （76例）	>65岁组 （55例）	统计量	*P*值
性别［例（％）］				0.162	0.922
男	27（47.4）	35（46.1）	24（43.6）		
女	30（52.6）	41（53.9）	31（56.4）		
体质性症状［例（％）］				3.999	0.135
有	47（82.5）	59（77.6）	50（90.9）		
无	10（17.5）	17（22.4）	5（9.1）		
基础合并症［例（％）］				18.933	<0.001
有	14（24.6）	35（46.1）	36（65.5）		
无	43（75.4）	41（53.9）	19（34.5）		
脾脏肋缘下长度［cm，*M*（*P*25，*P*75）］	10.0（6.0,14.0）	7.0（5.0,12.0）	9.5（4.3,12.0）	1.632	0.198
肝大［例（％）］				0.134	0.935
有	6（10.5）	9（11.8）	7（12.7）		
无	51（89.5）	67（88.2）	48（87.3）		
MPN-10症状评分总分［*M*（*P*25，*P*75）］	20.0（13.0,27.5）	23.5（15.0,30.0）	30.5（19.3,37.0）	6.204	0.002
血常规［*M*（*P*25，*P*75）］					
WBC（×10^9^/L）	14.1（6.4,18.1）	12.5（6.5,19.5）	17.0（9.0,24.7）	3.378	0.036
HGB（g/L）	109.0（92.5,128.5）	100.0（84.0,121.0）	98.0（76.0,118.0）	0.762	0.468
PLT（×10^9^/L）	324.0（190.0,425.5）	306.5（170.8,442.8）	267.0（179.0,368.0）	0.044	0.957
骨髓原始细胞比例［例（％）］				3.966	0.138
<1％	52（91.2）	60（78.9）	44（80.0）		
≥1％	5（8.8）	16（21.1）	11（20.0）		
外周血原始细胞比例［例（％）］				1.466	0.480
<1％	52（91.2）	66（86.8）	46（83.6）		
≥1％	5（8.8）	10（13.2）	9（16.4）		
骨髓纤维化等级［例（％）］				6.945	0.031
<2	11（19.3）	4（5.3）	5（9.1）		
≥2	46（80.7）	72（94.7）	50（90.9）		
驱动基因突变类型［例（％）］				15.213	0.012
JAK2	43（75.4）	57（75.0）	49（89.1）		
CALR	13（22.8）	14（18.4）	2（3.6）		
MPL	0（0.0）	4（5.3）	2（3.6）		
三阴型	1（1.8）	0（0.0）	1（1.8）		
双突变	0（0.0）	1（1.3）	1（1.8）		
输血依赖［例（％）］				5.332	0.070
有	4（7.0）	7（9.2）	11（20.0）		
无	53（93.0）	69（90.8）	44（80.0）		
骨髓纤维化亚型［例（％）］				1.683	0.808
PMF	45（78.9）	57（75.0）	39（70.9）		
PPV-MF	3（5.3）	5（6.6）	6（10.9）		
PET-MF	9（15.8）	14（18.4）	10（18.2）		
IPSS评分［例（％）］				73.591	<0.001
低危	7（12.3）	8（10.5）	0（0.0）		
中危-1	30（52.6）	31（40.8）	2（3.6）		
中危-2	16（28.1）	27（35.5）	17（30.9）		
高危	4（7.0）	10（13.2）	36（65.5）		
DIPSS评分［例（％）］				23.108	<0.001
低危	7（12.3）	6（7.9）	0（0.0）		
中危-1	33（57.9）	32（42.1）	19（34.5）		
中危-2	17（29.8）	30（39.5）	28（50.9）		
高危	0（0.0）	8（10.5）	8（14.5）		

**注** PMF：原发性骨髓纤维化；PPV-MF：真性红细胞增多症后骨髓纤维化；PET-MF：原发性血小板增多症后骨髓纤维化；IPSS：国际预后积分系统；DIPSS：动态国际预后积分系统

二、疗效评估

159例患者可评估脾脏大小变化，80.5％的患者在用药后的任何时间出现脾肿大改善，左肋缘下可触及脾脏长度较基线值中位减少42.9％。三组患者脾肿大改善程度差异无统计学意义（*P*＝0.530）。其中，≤55岁组83.0％（44/53）的患者用药后出现脾肿大改善，中位降低值为50.0％；56～65岁组79.0％（49/62）的患者用药后出现脾肿大改善，中位降低值为41.3％；>65岁组79.5％（35/44）的患者用药后出现脾肿大改善，中位降低值为43.9％。三组患者分别有60.4％（32/53）、54.8％（34/62）、59.1％（26/44）获得左肋缘下可触及脾脏长度较基线减少≥35％（*P*＝0.820）；50.9％（27/53）、43.5％（27/62）、45.5％（20/44）获得左肋缘下可触及脾脏长度较基线减少≥50％（*P*＝0.720）。

169例可评估MPN-10症状评分总分的患者中，83.4％的患者MPN-10评分较基线降低，中位降低值为57.9％。三组患者MPN-10评分降低值差异无统计学意义（*P*＝0.153）。≤55岁组中76.0％（38/50）的患者MPN-10评分较基线降低，中位降低值为50.0％；56～65岁组80.9％（55/68）的患者MPN-10评分较基线降低，中位降低值为56.2％；>65岁组94.1％（48/51）的患者MPN-10评分较基线降低，中位降低值为68.2％。三组分别有54.0％（27/50）、60.3％（41/68）、66.7％（34/51）的患者获得TSS50（*P*＝0.429）。

三、染色体核型及基因突变分析

100例患者在接受芦可替尼治疗前进行了染色体核型分析，109例患者在接受芦可替尼治疗前进行了高通量测序技术（NGS）检测（表2）。在染色体核型方面，≤55、56～65、>65岁组分别有12.9％、29.7％、21.9％的患者检出染色体核型异常（*P*＝0.249），分别有6.5％、10.8％、9.4％的患者存在预后不良核型（*P*＝0.909），包括复杂核型、+8、−7/7q−、i（17q）、−5/5q−、12p−、inv（3）或11q23重排的单个或两个异常。

**表2:** 芦可替尼治疗的不同年龄组骨髓纤维化患者细胞遗传学和分子学特征

临床特征	≤55岁组	56～65岁组	>65岁组	统计量	*P*值
染色体核型异常［例（％）］				2.784	0.249
有	4（12.9）	11（29.7）	7（21.9）		
无	27（87.1）	26（70.3）	25（78.1）		
预后不良核型［例（％）］				0.473	0.909
有	2（6.5）	4（10.8）	3（9.4）		
无	29（93.5）	33（89.2）	29（90.6）		
JAK2突变［例（％）］				8.180	0.017
有	21（67.7）	31（72.1）	33（94.3）		
无	10（32.3）	12（27.9）	2（5.7）		
JAK2V617F VAF［*M*（*P*_25_，*P*_75_）］	42.0（0,86.5）	48.6（0,81.4）	86.9（43.9,93.5）	5.698	0.005
CALR1型突变［例（％）］				5.171	0.071
有	6（19.4）	7（16.3）	1（2.9）		
无	25（80.6）	36（83.7）	34（97.1）		
非驱动基因突变［例（％）］				7.829	0.020
有	21（67.7）	40（93.0）	27（77.1）		
无	10（32.3）	3（7.0）	8（22.9）		
HMR突变［例（％）］				2.406	0.300
有	14（45.2）	27（62.8）	18（51.4）		
无	17（54.8）	16（37.2）	17（48.6）		
≥2个HMR突变［例（％）］				1.090	0.580
是	4（12.9）	8（18.6）	8（22.9）		
否	27（87.1）	35（81.4）	27（77.1）		

**注** VAF：变异等位基因频率，HMR突变：高分子风险突变

≤55、56～65、>65岁组分别有67.7％、72.1％、94.3％的患者检出JAK2V617F突变（*P*＝0.017），JAK2V617F变异等位基因频率（variant allele frequency, VAF）中位数分别为42.0％、48.6％、86.9％（*P*＝0.005）。组间比较结果显示，>65岁老年患者的JAK2V617F VAF较高（与≤55岁组比较，*P*＝0.023；与56～65岁组比较，*P*＝0.005），表明65岁以上老年MF患者JAK2V617F突变负荷较重。

≤55、56～65、>65岁组分别有21例（67.7％）、40例（93.0％）、27例（77.1％）患者检出非驱动基因突变，其中56～65岁组患者非驱动基因突变发生率较高（*P*＝0.02）。如图1所示，≤55岁组最常见的突变基因为ASXL1（41.9％）、TP53（9.7％）、TET2（9.7％）、NF1（9.7％）、SRSF2（6.5％）、SF3B1（6.5％）、U2AF1（6.5％）、NRAS（6.5％）；56～65岁组最常见的突变基因为ASXL1（48.8％）、TET2（14.0％）、SRSF2（14.0％）、DNMT3A（14.0％）、TP53（11.6％）、SF3B1（9.3％）、EZH2（7.0％）、U2AF1（7.0％）、BCORL1（7.0％）；>65岁组最常见的突变基因为ASXL1（40.0％）、TET2（28.6％）、SRSF2（14.3％）、SF3B1（11.4％）、TP53（8.6％）、DNMT3A（8.6％）、IDH2（8.6％）、EZH2（8.6％）、SH2B3（8.6％）、NF1（5.7％）。随年龄增加，IDH2、EZH2、TET2、SRSF2、SF3B1基因突变率增加，但差异无统计学意义。

**图1:**
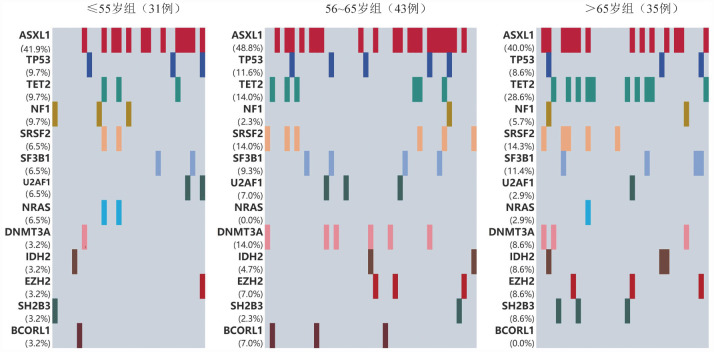
不同年龄组骨髓纤维化患者常见非驱动基因突变谱

四、生存与预后因素分析

截至末次随访，188例患者中共有27例患者死亡，死亡原因包括急性白血病进展16例（59.3％）、感染3例（11.1％）、多系统器官功能衰竭2例（7.4％）、脑出血1例（3.7％）、原因不明5例（18.5％）。截至末次随访时间，总体中位随访时间为27.8个月，中位生存时间未达到。其中，≤55岁组患者的中位生存时间未达到，56～65岁组中位OS期为116.2（95％ *CI* 10.4～222）个月，>65岁组中位OS期为66.4（95％ *CI* 47.0～85.8）个月，三组OS期差异具有统计学意义（*P*＝0.03）。生存曲线见图2。

**图2:**
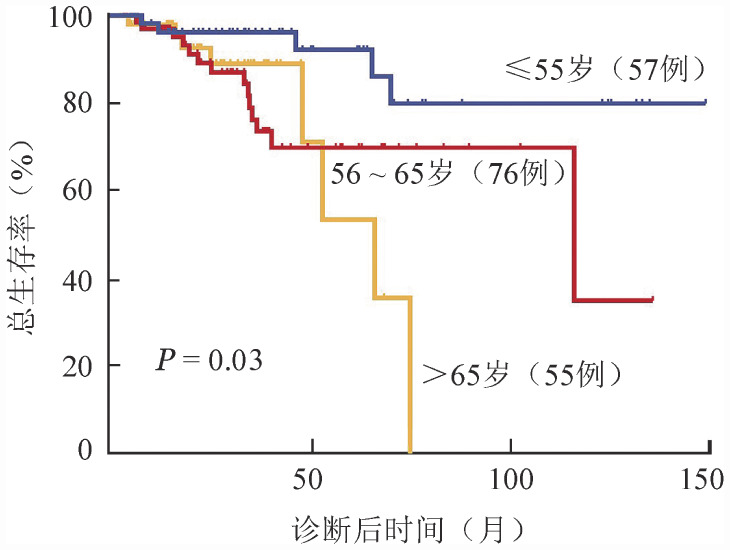
芦可替尼治疗的不同年龄组骨髓纤维化患者总生存曲线

对所有接受芦可替尼治疗的MF患者队列进行OS的单因素分析，年龄增加、MPN-10评分高、血红蛋白值降低、骨髓原始细胞比例升高、外周血原始细胞比例升高、无JAK2突变、具有CALR1型突变、有输血依赖性与患者OS缩短有关（*P*<0.05）。将上述变量纳入多因素分析，年龄增加、血红蛋白值降低、骨髓原始细胞比例≥1％、无JAK2突变是影响患者生存预后的独立危险因素（*P*<0.05）（表3）。

**表3:** 基线临床特征对接受芦可替尼治疗骨髓纤维化患者总生存影响的单因素及多因素分析

变量	单因素分析		多因素分析	
	*P*值	风险比（95%*CI*）	*P*值	风险比（95%*CI*）
性别（女）	0.058	0.47（0.21~1.03）		
年龄	0.006	1.06（1.02~1.11）	0.020	1.06（1.01~1.12）
体质性症状	0.384	1.90（0.45~8.06）		
基础合并症	0.715	1.15（0.54~2.47）		
脾脏肋缘下可触及	0.443	0.97（0.90~1.05）		
肝大	0.057	2.31（0.97~5.48）		
MPN-10总分	0.007	1.04（1.01~1.07）	0.135	1.03（0.99~1.06）
白细胞计数	0.100	1.01（1.00~1.03）		
血红蛋白浓度	0.005	0.98（0.97~0.99）	0.034	0.98（0.97~1.00）
血小板计数	0.061	1.00（1.00~1.00）		
骨髓原始细胞≥1%	0.003	3.71（1.58~8.71）	0.029	3.90（1.15~13.18）
外周血原始细胞≥1%	0.007	3.31（1.30~7.38）	0.975	0.98（0.27~3.52）
骨髓纤维化等级≥2	0.907	0.93（0.28~3.11）		
JAK2突变	0.002	0.29（0.13~7.38）	0.019	0.21（0.06~0.77）
CALR-1型突变	0.011	3.10（1.30~7.38）	0.714	0.77（0.18~3.21）
MPL突变	0.054	4.28（0.98~18.77）		
输血依赖	0.038	2.63（1.05~6.56）	0.460	0.66（0.22~1.98）

**注** MPN-10：骨髓增殖性肿瘤总症状评估量表

对接受芦可替尼治疗前进行了NGS检测的MF患者队列进行细胞遗传学和分子学相关的OS的单因素分析，存在染色体异常、存在预后不良核型、JAK2V617F VAF降低、CALR VAF升高、≥3个非驱动突变、≥2个HMR突变、TP53突变、NRAS突变与患者OS缩短有关（*P*<0.05）。将上述变量纳入多因素分析，存在染色体核型异常、≥2个HMR突变、TP53突变是影响患者生存预后的独立危险因素（*P*<0.05）（表4）。

**表4:** 细胞遗传学和分子特征对接受芦可替尼治疗骨髓纤维化患者总生存影响的单因素及多因素分析

变量	单因素分析		多因素分析	
	*P*值	风险比（95%*CI*）	*P*值	风险比（95%*CI*）
染色体异常	0.003	4.24（1.65~10.90）	0.014	6.55（1.46~29.37）
预后不良核型	<0.001	7.99（2.53~25.22）	0.826	1.23（0.19~7.87）
JAK2V617F VAF	0.039	0.99（0.98~1.00）	0.304	0.99（0.97~1.01）
CLAR VAF	0.011	1.03（1.01~1.05）	0.232	1.02（0.99~1.06）
MPL VAF	0.086	1.03（1.00~1.05）		
存在非驱动突变	0.819	1.14（0.38~3.39）		
≥3个非驱动突变	0.026	2.66（1.12~6.32）	0.963	1.03（0.26~4.03）
存在HMR突变	0.290	1.61（0.67~3.89）		
≥2个HMR突变	0.004	3.64（1.52~8.69）	0.019	4.12（1.26~13.46）
ASXL1突变	0.057	2.33（0.98~5.59）		
TP53突变	0.017	5.06（1.34~19.02）	0.048	5.15（1.02~26.10）
TET2突变	0.078	2.19（0.92~5.26）		
SRSF2突变	0.411	1.59（0.53~4.78）		
SF3B1突变	0.485	0.49（0.07~3.65）		
JDH2突变	0.572	0.56（0.07~4.18）		
EZH2突变	0.088	3.69（0.82~16.50）		
U2AF1突变	0.057	4.32（0.96~19.46）		
NRAS突变	0.033	3.92（1.12~13.73）	0.237	2.93（0.49~17.40）
SH2B3突变	0.270	0.30（0.03~2.56）		
BCORL1突变	0.390	2.45（0.32~18.91）		

**注** VAF：变异等位基因频率；HMR：高分子风险

五、安全性

对所有接受芦可替尼治疗的MF患者进行了安全性评估（表5），168例（89.4％）患者发生≥1次不良反应。≤55、56～65、>65岁组不良反应发生率分别为82.5％、92.1％、92.7％。常见的血液学不良反应为贫血135例（71.8％）、血小板减少70例（37.2％），常见的非血液学不良反应为电解质紊乱53例（28.2％）、转氨酶升高40例（21.3％）、肺部感染36例（19.1％）。94例（50.0％）患者发生1次及以上≥3级的严重不良反应，76.6％为血液学严重不良反应，8.5％为非血液学严重不良反应，14.9％两者均有发生。其中，≤55、56～65、>65岁组严重不良反应发生率分别为45.6％、48.7％、56.4％，差异无统计学意义（*P*＝0.501）。

**表5:** 188例接受芦可替尼治疗骨髓纤维化患者的治疗相关不良反应［例（％）］

不良反应	总体（188例）	年龄分组		
		≤55岁（57例）	56-65岁（76例）	>65岁（55例）
血液学不良反应				
贫血				
所有级别	135（71.8）	37（64.9）	52（68.4）	46（83.6）
≥Ⅲ级	72（38.3）	20（35.1）	25（32.9）	27（49.1）
血小板减少				
所有级别	70（37.2）	24（42.1）	25（32.9）	21（38.2）
≥Ⅲ级	37（19.7）	11（19.3）	14（18.4）	12（21.8）
白细胞减少				
所有级别	28（14.9）	10（17.5）	13（17.1）	5（9.1）
≥Ⅲ级	12（6.4）	4（7.0）	5（6.6）	3（5.5）
非血液学不良反应				
电解质紊乱				
所有级别	52（27.7）	15（26.3）	14（18.4）	23（41.8）
≥Ⅲ级	5（2.7）	2（3.5）	1（1.3）	2（3.6）
转氨酶升高				
所有级别	40（21.3）	11（19.3）	19（25.0）	10（18.2）
≥Ⅲ级	0	0	0	0
肺部感染				
所有级别	36（19.1）	8（14.0）	17（22.4）	11（20.0）
≥Ⅲ级	20（10.6）	6（10.5）	8（10.5）	6（10.9）
其他感染				
所有级别	9（4.8）	3（5.3）	6（7.9）	0
≥Ⅲ级	2（1.1）	0	2（2.6）	0
胆红素升高				
所有级别	26（13.8）	9（15.8）	7（9.2）	10（18.2）
≥Ⅲ级	1（0.5）	1（1.8）	0	0
肌酐升高				
所有级别	11（5.9）	4（7.0）	3（3.9）	4（7.3）
≥Ⅲ级	1（0.5）	1（1.8）	0	0
心血管事件				
所有级别	36（19.1）	7（12.3）	16（21.1）	13（23.6）
≥Ⅲ级	3（1.6）	0	1（1.3）	2（3.6）
胃肠道反应				
所有级别	12（6.4）	1（1.8）	4（5.3）	7（12.7）
≥Ⅲ级	0	0	0	0
神经系统不良反应				
所有级别	9（4.8）	3（5.3）	5（6.6）	1（1.8）
≥Ⅲ级	0	0	0	0
出血性疾病				
所有级别	12（6.4）	3（5.3）	6（7.9）	3（5.5）
≥Ⅲ级	0	0	0	0
发热				
所有级别	11（5.9）	3（5.3）	3（3.9）	5（9.1）
≥Ⅲ级	0	0	0	0

## 讨论

MF的临床表现和分子学特征具有广泛异质性[8]。年龄是骨髓纤维化IPSS、DIPSS、DIPSS-plus预后评分系统中的变量，有研究发现，年龄的增长与MF患者突变发生率的增加相关，高危基因在老年患者中较为常见，且一些高危基因的突变负荷随着年龄的增长而增加[4],[9]。基于此，本研究以55、65岁为年龄界限，将患者分为≤55、56～65及>65岁三个年龄组，对比分析不同年龄组患者临床特征和基因突变的异质性。我们观察到>65岁组患者基础合并症较多、治疗前症状负荷较重、白细胞计数较高、JAK2基因突变类型占比及突变频率较高、CALR基因突变类型占比较低。这与既往研究中CALR突变的MF患者通常较年轻，JAK2突变的MF患者的白细胞计数高于MPL和CALR突变的患者的发现是一致的[9]。然而，我们并没有观察到突变发生率、高危基因突变率随年龄的增长而增加，在本研究中，56～65岁组患者非驱动基因突变发生率显著较高，不同年龄组患者常见非驱动基因突变谱无显著差异。在过去的十余年中，JAK抑制剂已成为MF的关键治疗药物，目前已有芦可替尼、菲达替尼、帕瑞替尼、莫洛替尼等多种JAK抑制剂[10]。芦可替尼在2011年获批后目前仍然是许多具有脾大和（或）全身症状MF患者的一线治疗，也是目前国内唯一一种临床可用的JAK抑制剂。随着近年来相关研究数据的积累，芦可替尼越来越多地应用于65岁以上老年患者。鉴于不同年龄组MF患者临床特点和分子学特征存在一定的异质性，本研究对比分析了三组患者接受芦可替尼治疗后的疗效和安全性。在经典COMFORTⅠ试验中，芦可替尼治疗组中有41.9％的患者在24周时获得脾脏体积较基线缩小≥35％（SVR35），45.9％获得TSS50[6]。在本研究中，≤55、56～65、>65岁组分别有60.4％、54.8％、59.1％的患者在用药后的任何时间获得左肋缘下可触及脾脏长度较基线减少≥35％，50.9％、43.5％、45.5％的患者获得左肋缘下可触及脾脏长度较基线减少≥50％，54.0％、60.3％、66.7％的患者获得TSS50。总体而言，在现实世界中，芦可替尼在改善脾肿大和降低症状负荷方面表现出相当好的疗效。其中，55岁及以下中年患者脾肿大改善比例和程度较高，65岁以上老年患者骨髓纤维化相关症状改善的比例和程度较高，但总体三组患者脾脏缩小和MPN-10症状评分总分降低程度无显著差异。

在安全性方面，与既往报道一致，最常见的血液学不良反应为贫血和血小板减少，非血液学不良反应为电解质紊乱、转氨酶升高和肺部感染，可通过剂量调整进行管理，很少需要停用芦可替尼[11]–[13]。相较于中年患者，老年患者不良反应发生率增加，但总体三组患者不良反应发生率无显著差异。由此可见，虽然65岁以上老年患者基础合并症多、症状负荷重、白细胞计数高、JAK2V617F突变负荷重，但接受芦可替尼治疗后三组患者的疗效和不良反应发生率差异无统计学意义。因此，高龄不应成为芦可替尼治疗的限制因素。

MF的当代预后分层模型始于2009年IPSS的开发，纳入了5个临床风险预测因素[2]。随后开发了DIPSS，可在疾病过程中的任何时间应用[14]。MF的最新预后模型MIPSS70、MIPSS70+ 2.0在临床风险因素的基础上结合了基因突变和细胞遗传学信息，可能有助于更准确地对MF患者进行预后评估[15]–[17]。ASXL1、EZH2、IDH1、IDH2、SRSF2、U2AF1Q157已被证明推动疾病进展和急性白血病转化，被归类为HMR突变[9]，新预后评分系统强调了HMR突变对MF患者的预后意义。通过对本研究中患者的非驱动基因突变谱进行分析，我们发现HMR突变在不同年龄组患者中突变率均较高，可见新预后评分系统在实际临床实践中具有重要而广泛的应用价值。此外，MF的预后模型主要用于治疗决策，目前，预测芦可替尼治疗对生存的影响及造血干细胞移植的最佳时机是公认的未满足的医疗需求[18]–[19]。为了在真实世界中确定芦可替尼治疗的MF患者生存率降低的早期预测因素，本研究对所有接受芦可替尼治疗的MF患者队列进行OS的单因素分析和多因素分析。我们发现，基于临床特征因素，年龄增加、血红蛋白值降低、骨髓原始细胞比例≥1％、无JAK2突变是患者OS的独立危险因素；基于细胞遗传学和分子学因素，存在染色体核型异常、≥2个HMR突变、TP53突变阳性是患者OS的独立危险因素。未来，随着新药的研发和治疗方法的进展，根据每个患者的临床和遗传特征设计个性化治疗方案，可能会最大限度地改善疾病进程和结局[17],[20]。

综上所述，虽然不同年龄组接受芦可替尼治疗的MF患者的临床特征、基因突变具有异质性，但治疗后脾脏缩小和MPN-10症状评分总分降低程度无显著差异，具有相当的疗效和不良反应发生率。因此，对于具有治疗适应证的老年患者，芦可替尼有效且安全性良好。
